# IgE-binding monocytes upregulate the coagulation cascade in allergic horses

**DOI:** 10.1038/s41435-023-00207-w

**Published:** 2023-05-16

**Authors:** Elisabeth M. Simonin, Bettina Wagner

**Affiliations:** Department of Population Medicine and Diagnostic Sciences, College of Veterinary Medicine, Cornell University, Ithaca, NY USA

**Keywords:** Monocytes and macrophages, RNA sequencing

## Abstract

IgE-binding monocytes are a rare peripheral immune cell type involved in the allergic response through binding of IgE on their surface. IgE-binding monocytes are present in both healthy and allergic individuals. We performed RNA sequencing to ask how the function of IgE-binding monocytes differs in the context of allergy. Using a large animal model of allergy, equine *Culicoides* hypersensitivity, we compared the transcriptome of IgE-binding monocytes in allergic and non-allergic horses at two seasonal timepoints: (i) when allergic animals were clinical healthy, in the winter “Remission Phase”, and (ii) during chronic disease, in the summer “Clinical Phase”. Most transcriptional differences between allergic and non-allergic horses occurred only during the “Remission Phase”, suggesting principal differences in monocyte function even in the absence of allergen exposure. *F13A1*, a subunit of fibrinoligase, was significantly upregulated at both timepoints in allergic horses. This suggested a role for increased fibrin deposition in the coagulation cascade to promote allergic inflammation. IgE-binding monocytes also downregulated *CCR10* expression in allergic horses during the “Clinical Phase”, suggesting a defect in maintenance of skin homeostasis, which further promotes allergic inflammation. Together, this transcriptional analysis provides valuable clues into the mechanisms used by IgE-binding monocytes in allergic individuals.

## Introduction

IgE-mediated allergies are caused by the binding of allergen-specific IgE to receptors on basophils and mast cells, which degranulate when exposed to allergen [1, 2]. IgE-binding monocytes can also bind IgE through a variant of FcεRI [3–6] and circulate in allergic and healthy individuals [3]. IgE-binding monocytes provide innate protection from a broad range of pathogens and danger signals, and can respond to allergen through FcεRI-bound IgE. However, the role of IgE-binding monocytes in promoting allergy is still being understood. We previously showed that IgE-binding monocytes are more capable of IL-8 production in allergic than healthy horses, suggesting a pro-inflammatory bias in allergic individuals through enhanced recruitment of basophils and neutrophils by IL-8 [7].

Monocytes are a heterogenous cell population that can adopt a wide range of functions, depending on distinct developmental pathways [8]. Monocyte fates are determined by the inflammatory environment of the individual and specific inflammatory signals can even alter monopoiesis in the bone marrow, affecting future monocyte populations [9]. Allergies develop in some individuals upon exposure to allergen and, through a cascade of events, become a recurrent disease with every subsequent allergen contact. We hypothesized that specific inflammatory responses in some individuals, and the resultant monocyte populations that arise, help predispose these individuals, and not others, to develop allergy.

In order to better understand the role of IgE-binding monocytes in allergy, we used a large animal model affected by an allergy called equine *Culicoides (Cul)* hypersensitivity [10–18], which is mechanistically similar to IgE-mediated allergies in humans [19, 20]. *Cul* hypersensitivity is an IgE-mediated allergy in horses [3, 21–27] caused by the inappropriate response to salivary proteins released during the bites of *Cul* midges [28–31]. *Cul* are present in the environment only during warm and humid months, resulting in a seasonal disease where allergic individuals have signs of allergy in the summer and appear clinically healthy in the winter. This model allows comparison of immune cells when the horses have clinical allergy or are in remission to determine how cells are programmed to remember and maintain allergy.

In this article, we purified IgE-binding monocytes from allergic and healthy individuals at two seasonal timepoints, the “Remission Phase” in the winter and the “Clinical Phase” in the summer. We performed RNA sequencing (RNA-seq) on IgE-binding monocytes from each phase to ask how the IgE-binding monocyte transcriptome differs between allergic and healthy horses. We also asked if certain pathways were more activated in IgE-binding monocytes from allergic horses, and whether these genes were always activated or only activated during one or the other seasonal timepoint. To our knowledge, this is the first transcriptional analysis of IgE-binding monocytes and comparison between allergic and healthy individuals.

## Materials and Methods

### Animals and blood collection

All animals included in this study were Icelandic horses with heterogenous genetic backgrounds, as determined previously by MHC haplotype [32]. All horses were annually vaccinated against rabies, tetanus, West Nile virus, Eastern and Western Encephalitis virus, and in December were dewormed with moxidectin and praziquantel (Zoetis, Parsipanny, NJ, USA). Vaccination and deworming were synchronized for all animals. Horses lived together full time on large pastures with run-in sheds, free access to water, mineral salt blocks, and were fed grass (summer) and grass hay (winter).

All horses also had similar exposure to *Cul* midges from mid-May to mid-October, and no exposure to midges during the winter months. Horses were either clinically healthy (*n* = 5) or had naturally occurring, seasonal *Cul* hypersensitivity (*n* = 7). The sample size was selected based on the previously reported prevalence of allergic disease for Icelandic horses that are exported from Iceland as adults (20–60% prevalence) [11, 33, 34] so that about half of the horses would be allergic. Allergic horses included 6 mares (ages 9–15, median 13.5) and 1 gelding (7 years), and healthy horses included 5 mares (5–12 years, median 6 years). Two additional healthy horses were excluded from the analysis because they developed signs of allergy after the study was completed. In January and August, heparinized blood was collected by *V. jugularis* venipuncture using a vacutainer system (Becton Dickinson, Franklin Lakes, NJ). Blood samples were used for peripheral blood mononuclear cell (PBMC) isolation followed by IgE-binding monocyte cell sorting and RNA-seq. Two-four horses were sampled each day over 7-weeks in January–February and 3-weeks in August-September. In January (“Remission Phase”), all horses were clinically healthy. In August (“Clinical Phase”), allergic horses had chronic, severe clinical signs of allergy, while healthy horses had no clinical signs (Table 1).

**Table 1:** Study population of allergic horses that only show clinical disease during allergen exposure in the “Clinical Phase” and healthy horses that never show clinical disease.

	“Remission Phase”^a^ Clinical Score, median (range)	“Clinical Phase”^b^ Clinical Score, median (range)
Allergic Group (*n* = 7)	0 (0–1)	6.5 (4–7)
Healthy Group (*n* = 5)	0 (0–0)	0 (0–2)

^a^There was no *Culicoides* allergen exposure in winter months. Scores were assigned in January and all horses were clinically healthy.

^b^All horses were chronically exposed to *Culicoides* allergen in summer months from June-September. Scores (0–10) were assigned in August when allergic horses had chronic signs of allergy. Scores ≥ 3 are allergic.

*Cul* hypersensitivity was diagnosed by clinical signs and confirmed by intradermal skin testing with *Cul* whole body extract (WBE; Stallergenes Greer Inc., Cambridge, MA, USA) where allergic horses had immediate skin reactions and healthy horses did not. Histamine and saline intradermal injections served as positive and negative controls, respectively. As previously described [35], all horses were given a clinical allergy score every 2–4 weeks. Scores were assigned for pruritis (0–3), alopecia (0–4) and dermatitis (0–3). No randomization was used to assign groups.

All animal procedures were carried out in accordance with the recommendation in the Guide for the Care and Use of Laboratory Animals of the National Institutes of Health. The animal protocol was approved by the Institutional Animal Care and Use Committee at Cornell University (protocol #2011–0011). The study also followed the Guide for Care and Use of Animals in Agricultural Research and Teaching.

### IgE-binding monocyte sorting

PBMC were isolated from cell-rich plasma of whole blood by density gradient centrifugation, as previously described [3, 7]. Briefly, leukocyte-rich plasma was layered on Ficoll (Ficoll-PaqueTM Plus, GE Healthcare, Piscataway, NJ) and centrifuged. The interphase, containing PBMC, was collected and washed twice with phosphate buffered solution (PBS, Fisher Scientific, Waltham, MA, USA), and then washed in 10 ml PBS-BSA (0.5%(w/v) BSA, 0.02%(w/v) NaN3, all from Sigma-Aldrich, St. Louis, MO, USA).

Following PBMC isolation, cells were immediately prepared for sorting as previously described [3]. Briefly, PBMC were first incubated with an anti-equine CD14 monoclonal antibody (mAb, clone 105, biontinylated) [36], followed by incubation with goat-anti-mouse IgG1-coated magnetic beads (Miltenyi Biotech, Auburn, CA), and the CD14+ fraction was collected over an LD column (Miltenyi Biotech, Auburn, CA). Two aliquots of 1 × 10^8^ PBMC were sorted in parallel for each horse and CD14+ fractions were pooled after collection. For fluorescence-activated cell sorting (FACS), CD14+ cells were further stained with antibodies against equine MHCII (clone cz11, conjugated with phycoerythrin) [37], IgE (clone 176, conjugated with Alexa Fluor 488) [21], CD16 (clone 9G5, conjugated with Alexa Fluor 647) [38], CD163 (clone BerMac3, conjugated with Alexa Fluor 647) [39], and a viability dye (ThermoFisher Scientific, Waltham, MA, USA), followed by secondary staining with streptavidin-PerCP-Cyanin5.5 (BioLegend, San Diego, CA, USA) to label biotinylated CD14 mAb. Alexa conjugation and biotinylation of mAbs were performed according to manufacturer’s protocols (ThermoFisher Scientific, Waltham, MA, USA). UltraComp beads and amine reactive beads (both from ThermoFisher Scientific, Waltham, MA, USA) were single stained as compensation. At each sorting step, aliquots were also collected to check for sorting efficiency by flow cytometry. All antibody staining steps were performed in PBS-BSA on ice. Anti-IgE mAb 176 was used for FACS because it binds IgE but has minimal receptor crosslinking ability [3, 7, 21, 24].

FACS was performed on a BD FACS Aria Fusion sorter at Cornell University’s Biotechnology Resource Center (BRC) Flow Cytometry core through an 85 µm nozzle at 25 psi. Live IgE-binding monocytes (CD14^low^/IgE + /MHCII^high^/CD16-/CD163- cells) were collected into cell culture medium (DMEM supplemented with 1%(v/v) non-essential amino acids, 2mM L-glutamine, 50 µM 2-mercaptoethanol, 50 µg/ml gentamicin, 100 U/ml penicillin, 100 µg/ml streptomycin, all from ThermoFisher Scientific, Waltham, MA, USA, and 10% FCS from Atlanta Biological, Flowery Branch, GA, USA) at room temperature (RT) and viability was confirmed by trypan blue exclusion (STable 1). Sorted IgE-binding monocytes were cryopreserved in ice-cold cell culture medium supplemented with 10% dimethyl sulfoxide (DMSO, Sigma-Aldrich, St. Louis, MO, USA) and stored in liquid nitrogen until processing for RNA extraction. Cell yield of sorted IgE-binding monocyte fractions ranged from 7.1 × 10^4^–4.3 × 10^5^ cells/sample (median 2.5 × 10^5^ cells, *n* = 24).

### RNA extraction and Illumina sequencing

Frozen IgE-binding monocytes were thawed at 37 °C and slowly resuspended into 10 ml PBS-FCS (PBS supplemented with 20% FCS and 2 mM EDTA (Boston BioProducts, Boston, NY, USA) at RT. Cells were pelleted at 100 x g, 5 min, RT and washed once in 1 ml PBS-FCS. Cells were resuspended in 250 µl PBS-FCS and 750 µl Trizol LS solution (Invitrogen, Waltham, MA, USA). Then, 200 µl chloroform (Fisher Scientific, Pittsburgh, PA, USA) was added. Tubes were vigorously shaken for 15 s and centrifuged at 16,000 x g, 15 min, 4 °C. The aqueous phase was collected and added to an additional 600 µl chloroform in Phase Lock Gel tubes (Quantabio, Beverly, MA, USA). To improve RNA recovery, 2 µl of a glycogen carrier (GlycoBlue, ThermoFisher Scientific, Waltham, MA, USA) was added. Last, 500 µl isopropanol was added and incubated on ice for 1 h, then washed three times in 75% ice-cold ethanol. All wash spins were performed at 16,000 x g, 10 min, 4 °C. Extracted RNA was air dried in a laminar flow hood at RT, then resuspended in 20 µl RNase-free water (Invitrogen, Waltham, MA, USA), and stored at −80 °C until submitted for sequencing.

RNA concentration was determined by High Sensitivity RNA Qubit assay (Invitrogen, Waltham, MA, USA) and RNA integrity was measured with a Fragment Analyzer (Advanced Analytical, Orangeburg, NY, USA). All samples were of good quality (RQN median 10, range 7.6–10) and had a median RNA concentration of 36 ng/µl (range 22–49.3 ng/µl) (STable 1) during both timepoints.

Cornell University’s Transcriptional Regulation and Expression (TREx) Facility prepared RNA libraries for Illumina® Sequencing. RNA was first poly-A selected with the NEBNext Poly(A) mRNA Magnetic Isolation Module (New England Biolabs, Ipswich, MA, USA) and TruSeq-barcoded RNA-seq libraries were generated using the NEBNext® Ultra™ II Directional RNA Library Prep kit (New England Biolabs, Ipswich, MA, USA). Libraries were sequenced on an Illumina instrument with a read length of 75 bp single end reads at a depth of 20 million reads per sample.

### RNA-seq analysis

Raw sequencing reads were initially processed by Cornell’s TREx Facility using pipelines built around ENCODE standards and practices. First, raw fastq files were processed with TrimGalore v0.6.0 to remove low quality bases and adaptor sequences. Reads were then mapped to the EquCab3.0 genome using STAR v2.7.0e [40]. Read count data was further analyzed using iDEP.96 (integrated Differential Expression and Pathway analysis). Counts data was transformed using EdgeR (log2(CPM + 4)) on all genes with a minimum of 0.5 counts per million (CPM) in at least 1 library. Samples from each phase were normalized separately.

Differentially expressed genes (DEGs) between allergic and healthy horses were identified by DESeq2 [41] and fold change (FC) values always describe the change in allergic horse samples over/under healthy horse samples. Significance was calculated by False Discovery Rate (FDR), referred to as adjusted *p*-value (p) throughout, and 39 genes had an adjusted p-value (FDR) < 0.05 at one or both timepoints. These genes were mapped in a heatmap using heatmap.2 and ggplot2 functions. Data was centered by subtracting the average expression level for each gene. Hierarchical clustering of genes was performed with Euclidean distance and average linkage. Samples were clustered by seasonal timepoint and allergy group.

### Statistical analysis

The data were not normally distributed as confirmed by D’Agostino and Pearson tests. Therefore, non-parametric tests were used for analysis. A Holm-Sidak multiple comparisons test compared clinical scores and sorting purities. RNA-seq counts data statistics were calculated by a Wald test in DESeq2 [41]. Investigators were not blinded during analysis. Unless otherwise specified, graphs plot individual values and group medians. *P*-values < 0.05 were considered significant. Graphs were generated with GraphPad Prism software version 9.0.0 (GraphPad Software Inc., La Jolla, CA, USA).

## Results

### IgE-binding monocytes were purified during both phases of allergy

**Fig. 1:**
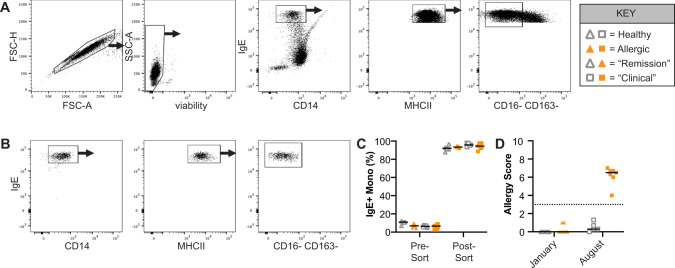
IgE-binding monocytes were purified from PBMC during clinical allergy and remission. PBMC were isolated from healthy (*n* = 5, gray Δ□) and allergic (*n* = 7, orange ▲■) horses in January (“Remission Phase”, triangles) and August (“Clinical Phase”, squares). IgE-binding monocytes were purified by magnetic CD14 enrichment followed by FACS. **A** Gating strategy to isolate IgE-binding monocytes from CD14-enriched cells. Images from left to right show doublet exclusion, viability selection, and gates on CD14^low^/IgE + , MHCII^high^, and CD16-/CD163- cells. **B** IgE-binding monocyte purity, after FACS, shown by IgE binding and CD14 expression (left), MHCII expression (middle) and lack of CD16/CD163 expression (right). **C** IgE-binding monocyte purities are summarized for all samples after CD14 enrichment (pre-sort) and after FACS (post-sort). **D** Clinical signs of allergic and healthy horses at both timepoints. Flow cytometry plots are representative from one horse in January.

IgE-binding monocytes were sorted from equine PBMC as previously described [3] from allergic (*n* = 7) and healthy (*n* = 5) horses. IgE-binding monocytes in equine peripheral blood are characterized as CD14^low^/IgE + /MHCII^high^/CD16-/CD163- cells (Fig. 1A). Total monocytes were first enriched by magnetic CD14+ sorting, then IgE-binding monocytes (CD14^low^/IgE + /MHCII^high^/CD16-/CD163-) were further purified by FACS (Fig. 1B). IgE-binding monocytes were enriched from 7.0% (median, range 2.4–11.9%) in CD14-enriched samples to 93.6% (median, range 87.8-97.8%) after FACS (Fig. 1C). The sample purities after FACS were calculated using a strict gating strategy, however even the cells outside of those gates still resemble IgE-binding monocytes but with marginally lower IgE binding or CD14 expression (Fig. 1B, left panel). IgE-binding monocytes were purified in January (“Remission Phase”) when allergic horses had no *Cul* allergen exposure or clinical signs of allergy, and in August (“Clinical Phase”) when horses were constantly exposed to environmental allergen and allergic horses experienced chronic clinical signs (Table 1, Fig. 1D). Healthy horses never experienced clinical allergy.

### IgE-binding monocytes exhibit distinct transcriptomes in allergic individuals during the “Remission Phase”

IgE-binding monocyte transcriptomes at both phases were then compared by RNA-seq. While the total raw read counts (SFigure 1A) varied between samples, after normalization the median counts distribution was consistent across samples, especially within each phase (SFigure 1B). During the “Remission Phase”, the normalized median counts distribution was 8.93 (mean, SD 0.055, *n* = 7) in allergic horses and 8.96 (mean, SD 0.008, *n* = 5) in healthy horses. During the “Clinical Phase”, the normalized median counts distribution was 7.94 (mean, SD 0.012, *n* = 7) in allergic horses and 7.92 (mean, SD 0.023, *n* = 5) in healthy horses. Principal component analysis revealed that IgE-binding monocytes from allergic and healthy horses had more distinct transcriptomes during the “Remission Phase” than the “Clinical Phase” (SFigure 1C).

**Fig. 2:**
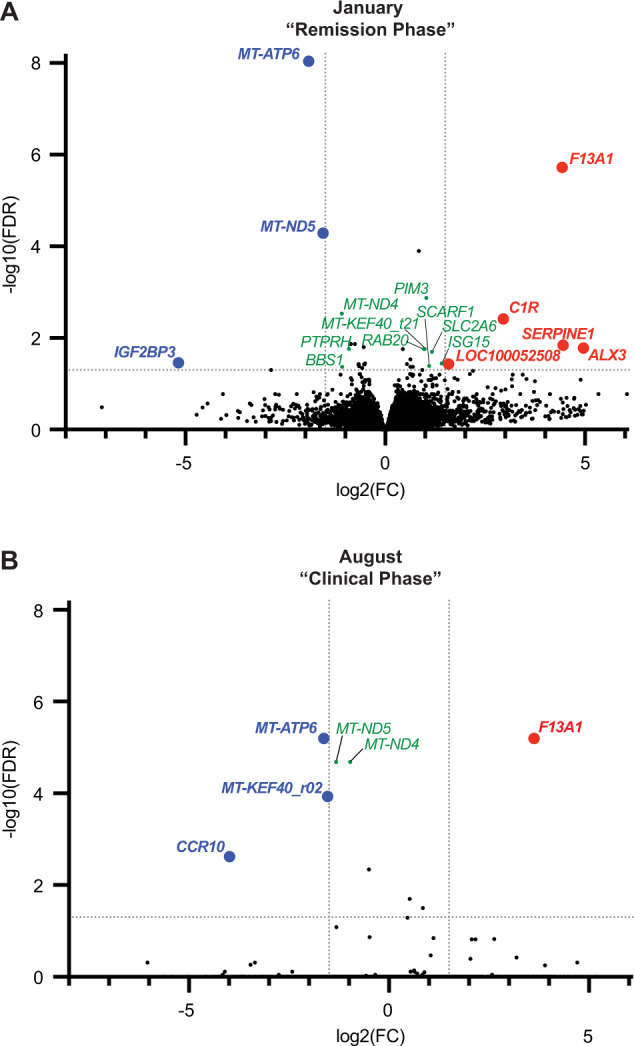
RNA-seq of IgE-binding monocytes reveals DEGs in allergic horses during both Remission and Clinical Phases. Normalized read counts were compared between healthy (*n* = 5) and allergic (*n* = 7) horses during the “Remission Phase” in January and the “Clinical Phase” in August. Volcano plots describe differentially expressed genes (DEGs) in allergic horses compared to healthy horses. Genes in the upper right and upper left regions are significantly up- (red) or downregulated (blue) in allergic horses, respectively. **A** DEGs during the “Remission Phase”. **B** DEGs during the “Clinical Phase”. Significant DEGs (*p* < 0.05) that were upregulated by > 1.5-fold change (red, positive log_2_(FC) values) or downregulated by > 1.5-fold change (blue, negative log_2_(FC) values) are labeled with corresponding gene names. Genes with +/− fold change (FC) values between 0.9 and 1.5 and *p* < 0.05 (green) are also labeled with gene name. Dotted lines have been added to aid visualization of DEGs. The horizontal dotted line shows the -log10 of the false discovery rate (FDR) threshold of 0.05 and values above this line are statistically significant. The vertical dotted lines show +/−1.5 FC in allergic compared to healthy horses.

Gene expression was then compared between groups at each allergy phase (Fig. 2). During the “Remission Phase”, five genes were upregulated and three genes were downregulated in allergic horses, all by at least 1.5 FC (*p* < 0.05, Fig. 2A), when compared to healthy horses. During the “Chronic Phase”, one gene was upregulated and three genes downregulated in allergic horses, all by at least 1.5 FC (*p* < 0.01, Fig. 2B), compared to healthy horses. IgE-binding monocytes in all horses had remarkably similar transcriptomes during the “Chronic Phase”, such that 99.8% of genes had no difference in expression between groups (*p* > 0.9 for 13 270/13 301 genes, Fig. 2B).

**Fig. 3:**
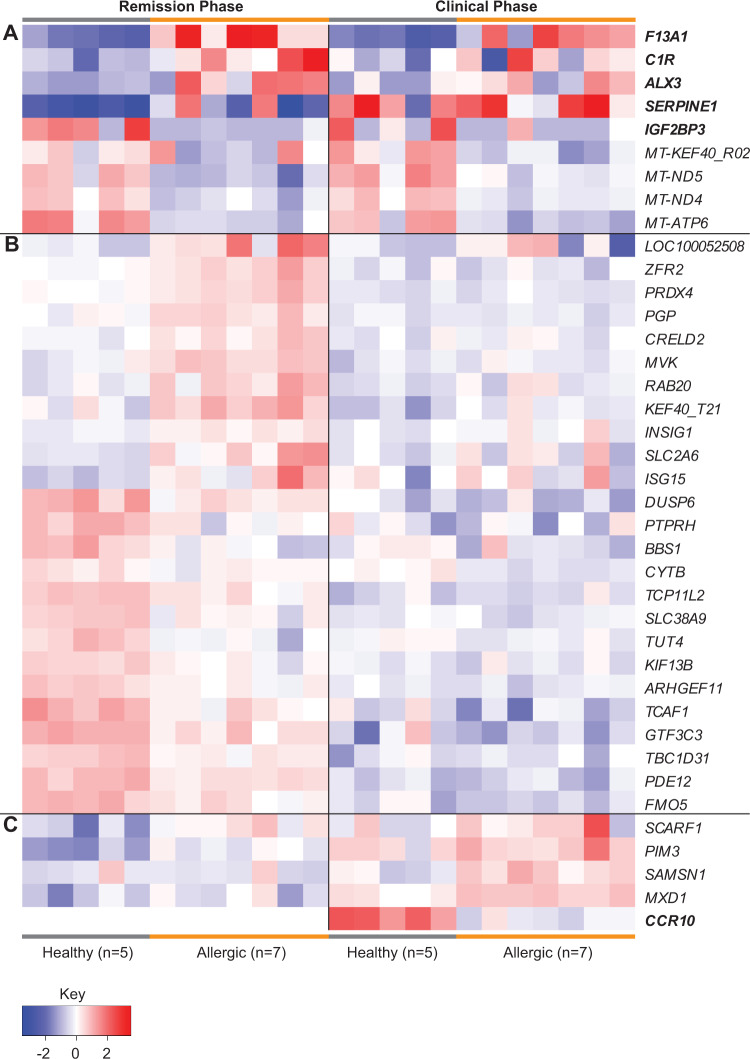
Heatmap of significant DEGs reveals gene expression clusters. A Heatmap was generated to show the expression of each DEG across individuals and at both timepoints. Each column represents a different horse, and samples are ordered healthy (*n* = 5, gray bars) then allergic (*n* = 7, orange bars) individuals for each phase. A vertical black line separates the “Remission Phase” (left) and “Clinical Phase” (right). Each row represents a different gene and gene expression is color coded to represent up- (red) or downregulation (blue) compared to the average for that gene. Genes have been grouped editorially to aid visualization of expression patterns: **A** DEGs in allergic horses at both timepoints, **B** DEGs in allergic horses only during the “Remission Phase”, and **C** DEGs in allergic horses only during the “Clinical Phase”. Genes with over +/−2 FC are bolded.

To further visualize differences in gene expression between IgE-binding monocytes from healthy and allergic horses, a heatmap was generated to show all the genes that were significantly differentially expressed at one or both timepoints (Fig. 3). All DEGs, regardless of FC, were included in the heatmap. The genes clustered in different expression patterns: (A) up- or downregulated in allergic horses at both timepoints, (B) up- or downregulated in allergic horses only during the “Remission Phase”, or (C) up- or downregulated in allergic horses only during the “Clinical Phase”.

One gene, *CCR10*, was primarily expressed by healthy horses only during the “Clinical Phase” with little to no expression in healthy horses during the “Remission Phase” or in allergic horses at either phase. During the “Clinical Phase”, healthy horses’ IgE-binding monocytes had 0.5–1.4 counts per million (CPM, *n* = 5) for *CCR10* transcripts while allergic horses had 0.1 CPM (*n* = 3), 0.2 CPM (*n* = 1) or no expression (*n* = 3) of *CCR10* transcripts. During the “Remission Phase”, *CCR10* was almost entirely unexpressed. Two healthy horses had 0.2–0.4 CPM and one allergic horse had 0.1 CPM of *CCR10* transcripts, but the remaining healthy (*n* = 3) and allergic (*n* = 6) horses had no *CCR10* expression during this phase. The counts values during the “Remission Phase” did not pass the threshold of 0.5 CPM during normalization and therefore *CCR10* was excluded from the “Remission Phase” analysis (Fig. 3C).

IgE-binding monocytes have previously been characterized to express CD14, IgE receptor subunits FcεRI alpha and gamma, IL-8 and IL-10 [3–7]. To further validate the accuracy of our RNA-seq results, expression of these key genes in IgE-binding monocytes were compared between healthy and allergic horses at each timepoint. As expected, IgE-binding monocytes in all horses expressed similar transcript levels for *CD14* and IgE receptor subunit genes *FCER1A* and *FCER1G* at both timepoints (SFigure 2A, B). IgE-binding monocytes also expressed transcripts for the IL-8 gene, *CXCL8*, and for the IL-10 gene, *IL10*, at both timepoints (SFigure 2A, B).

### IgE-binding monocyte transcriptional profiles suggest altered coagulation cascade and migratory ability in allergic horses

**Fig. 4:**
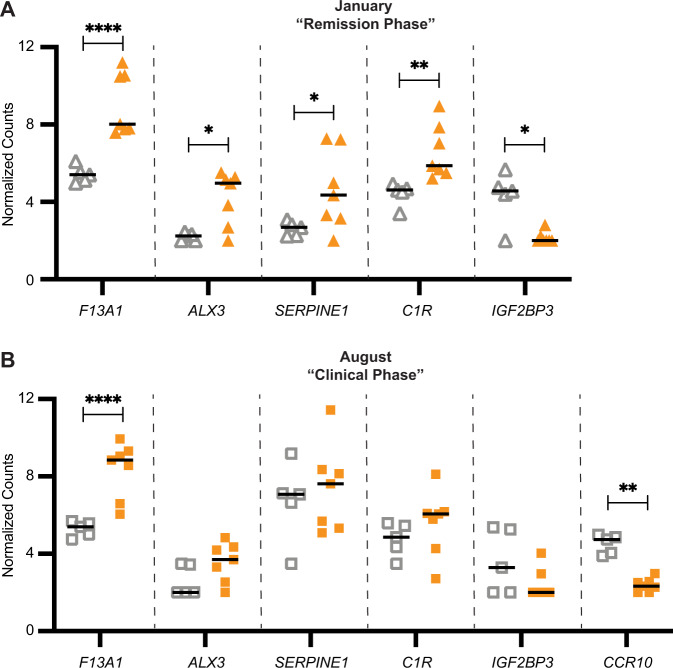
*F13A1*, *CCR10* and other genes are differentially expressed in allergic and healthy horses in Remission and Clinical Phases. Normalized read counts of DEGs with greater than +/− 2 FC were compared between healthy (*n* = 5, gray △□) and allergic (*n* = 7, orange ▲■) horses. These genes included *F13A1, ALX3, SERPINE1, C1R, IGF2BP3* and *CCR10*. **A** Read count comparison in January (“Remission Phase”, triangles). *CCR10* was not expressed at this timepoint and is excluded. **B** Read count comparison in August (“Clinical Phase”, squares). Graphs plot individual values and medians. *p < 0.05; **p < 0.01; ***p < 0.001, ****p < 0.0001.

To further explore transcriptional differences in IgE-binding monocytes at both allergy phases, genes that were up- or downregulated by more than 2 FC in allergic horses were further analyzed. Only one gene, *F13A1*, was significantly upregulated in allergic horses during both the “Remission Phase” (+4.4 FC, *p* = 0.0000019) and “Clinical Phase” (+3.5 FC, *p* = 0.0000064) (Fig. 4A, B). Three genes were only significantly upregulated in allergic horses during the “Remission Phase”: *ALX3* ( + 5 FC, *p* = 0.017), *SERPINE1* ( + 4.5 FC, *p* = 0.014) and *C1R* ( + 3 FC, *p* = 0.0039) (Fig. 4A). During the “Clinical Phase” (Fig. 4B), there was still a trend towards increased expression for *ALX3*, *SERPINE1* and *C1R* in allergic horses.

Two genes were downregulated in IgE-binding monocytes from allergic horses. *IGF2BP3* was downregulated during the “Remission Phase” (-5.2 FC, *p* = 0.035) (Fig. 4A) with only a downregulation trend during the “Clinical Phase”. *CCR10* was downregulated in allergic horses during the “Clinical Phase” (-4.0 FC, *p* = 0.0024) (Fig. 4B). IgE-binding monocytes did not express enough *CCR10* during the “Remission Phase” to be included in the DEG analysis, as described above. Three mitochondrial genes, ATP6, ND5 and KEF40, were also downregulated in allergic horses during one or both Phases (SFigure 3).

Together these differentially expressed immune system and mitochondrial genes suggest that IgE-binding monocytes exhibit different cell functions and regulatory capacities depending on the allergy status (allergic or healthy) and the phase of allergy (Remission or Clinical).

## Discussion

IgE-binding monocytes bridge innate and adaptive immune responses by responding to a broad range of molecular patterns and, through IgE, to specific allergens. We previously showed that IgE-binding monocytes exist at similar frequencies in healthy and allergic individuals [3], but produce more pro-inflammatory IL-8 in allergic individuals [7]. IgE-binding monocytes, therefore, have a potential role in the recruitment of allergy-associated cells and likely engage in different mechanisms in allergic and healthy individuals.

To find further evidence for the role of IgE-binding monocytes in allergy, we compared transcriptional profiles of these cells in allergic and healthy horses. *Cul* hypersensitivity is a naturally occurring, seasonal allergy in horses caused by *Cul* midge bites [10, 12, 13, 15, 18, 22, 25, 28, 29]. The mechanism of allergy, including the involvement of IgE-binding monocytes, is conserved between humans and horses [3–6, 19, 20]. All horses in this experimental approach were environmentally exposed to *Cul* allergens at the same time and it was therefore possible to compare immune cell function during both allergy phases.

While IgE-binding monocytes exhibited multiple up- and downregulated genes in allergic horses at both seasonal timepoints, most of the differential gene expression occurred in the winter. During the “Remission Phase”, horses were clinically healthy and had not been exposed to allergens in at least three months. Therefore, transcriptional differences at this timepoint were sustained in IgE-binding monocytes of allergic horses, regardless of allergen exposure and clinical disease, suggesting an altered baseline expression profile of IgE-binding monocytes in allergic individuals.

To validate the gene expression profile of our IgE-binding monocyte transcriptome dataset, we compared gene expression of cell-specific proteins that we previously characterized in horse IgE-binding monocytes. As expected [3], both allergic and healthy individuals had similar expression of transcripts for *CD14*, FcεRI alpha and gamma subunits, and *IL10*.

During the “Clinical Phase”, transcriptomes were similar between healthy and allergic horses, with only nine DEGs. This suggests that IgE-binding monocytes are remarkably similar in function and activation status in allergic and healthy horses during the summer. IgE-binding monocytes respond both to allergen, through IgE, and to pathogen-associated molecular patterns which are abundant in the saliva of many biting insects, through CD14 and other pattern recognition receptors [42]. As a result, IgE-binding monocytes in all horses likely were responding to a wide range of stimuli from environmental pressures in the summer, explaining the comparable transcriptional profiles between groups. Likewise, the overall few DEGs during the “Clinical Phase” suggest a stage of exhaustion in IgE-binding monocytes, possibly due to the summer’s manifold environmental triggers. However, these few DEGs in the “Clinical Phase” point to important roles in allergy-associated mechanistic modifications in IgE-binding monocytes from allergic horses. Each of these genes requires future follow up to determine how their encoded proteins are involved in the mechanism of IgE-binding monocytes in allergy.

One gene, *F13A1*, was significantly upregulated in allergic horse IgE-binding monocytes at both timepoints. *F13A1* encodes for coagulation factor XIIIa, a subunit of fibrinoligase. Fibrinoligase is a tetramer of two enzymatically active A subunits, encoded by *F13A1*, and two inhibitory B subunits [43], providing a self-regulatory mechanism to control the coagulation cascade. Overexpression of *F13A1* by alternatively activated macrophages, important for tissue remodeling and repair [44, 45], increases fibrin deposition and leads to pathogenesis. For example, in chronic rhinosinusitis patients, *F13A1* upregulation leads to increased fibrin in nasal polyps, thereby increasing disease severity [46]. One of the diagnostic signs of *Cul* hypersensitivity is dermatitis at sites of allergen exposure. Therefore, *F13A1* overexpression by IgE-binding monocytes of allergic individuals could induce excess fibrin buildup, as monocytes home to skin tissues to heal allergen*-*induced wounds, leading to dermatitis and scar tissue build-up typically seen in *Cul* hypersensitivity. Measuring *F13A1* expression in monocyte-derived cells in the skin of allergic horses will further support this hypothesis.

**Fig. 5:**
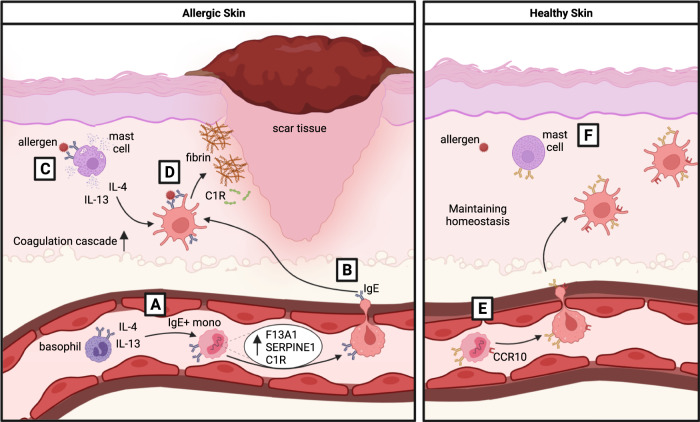
Proposed mechanism of F13A1 and the coagulation pathway in IgE-binding monocytes to promote allergic inflammation in the skin. IgE-binding monocytes (IgE+ Mono) circulate in peripheral blood. It is expected that these monocytes can home to the skin and we hypothesize that they help promote skin inflammation following allergen exposure in allergic individuals. **A** IgE-binding monocytes in peripheral blood of allergic individuals are triggered by increased levels of allergy-associated cytokines IL-4 and IL-13, likely from basophils. This upregulates *F13A1*. **B** IgE-binding monocytes, expressing *F13A1*, home to the skin. **C** In the skin, they are exposed to more IL-4 and IL-13 when mast cells degranulate following allergen exposure. *F13A1* expression increases further. **D** IgE-binding monocytes, which will differentiate into macrophages or dendritic cells, upregulate the coagulation cascade and deposit excess fibrin and C1R, ultimately leading to scar tissue build-up and exacerbating allergic wounds while also potentially using complement to guard against any additional microbes that may enter the broken skin. **E** Meanwhile, in healthy individuals, IgE-binding monocytes are not exposed to IL-4 and IL-13, do not upregulate *F13A1* or the coagulation cascade, and respond appropriately to allergen by not promoting skin inflammation. IgE-binding monocytes also upregulate *CCR10*, suggesting a mechanism for homing to healthy skin. **F** In addition, mast cells do not degranulate in tissues and homeostasis is maintained. Image was created on biorender.com.

Whether *F13A1* helps in causing allergic skin inflammation, or is upregulated in response to allergic wounds, also remains to be explored. *F13A1* in humans is upregulated by IL-4 or IL-13 stimulation [44, 47], which are responsible for B cell class switching to IgE [48–50]. Therefore, *F13A1* expression in IgE-binding monocytes could be induced in allergic individuals shortly after, or simultaneously with, the first development of allergen-specific IgE, when IL-4 and IL-13 are abundant [48]. Subsequent exposure to allergen and activation of peripheral basophils, which degranulate and release IL-4 [1, 23, 27], would further induce expression of *F13A1* in IgE-binding monocytes, setting up their elevated baseline expression in allergic horses (Fig. 5).

Similar to *F13A1*, *SERPINE1* and *C1R* are also upregulated in allergic horses, but only during the “Remission Phase”. There is a trend towards upregulation of both genes during the “Clinical Phase”. *SERPINE1* encodes a serine protease inhibitor, also known as plasminogen activating inhibitor 1 (PAI-1), that inhibits fibrinolysis [51, 52], thereby increasing fibrin deposition. PAI-1 is typically present in excess in circulation to regulate the coagulation cascade [53]. *C1R* is a proteolytic subunit of the classical complement system [54], and is often activated simultaneously with the coagulation pathway. *C1R*, along with other complement subunits, was found to be a substrate for *F13A1* [55]. Together, these genes emphasize the importance of the coagulation pathway in skin-localized allergy (Fig. 5). Whether allergic wounds induce upregulation of *F13A1*, *SERPINE1*, and *C1R*, or vis versa, is worth exploring.

Another gene that may be important in the allergy-associated role of IgE-binding monocytes is *CCR10*. This gene was downregulated in IgE-binding monocytes from allergic horses in the summer and hardly expressed by any horse in the winter. CCR10 is a critical regulator of skin immune homeostasis through binding of chemokine CCL27, produced by skin keratinocytes [56]. The CCL27/CCR10 axis has been suggested to promote allergic skin inflammation through recruitment of CCR10 + T-cells [57–59]. In addition, equine keratinocytes from allergic horses express *CCL27* transcripts following stimulation with *Cul* allergens [60]. However, here allergic horses downregulated *CCR10* expression in IgE-binding monocytes during allergen exposure. *CCR10* expression by IgE-binding monocytes requires further confirmation and may play a role in homing of these cells to the affected skin areas during allergen exposure. The role of this receptor on IgE-binding monocytes of healthy individuals will also be worth exploring.

CCR10 has been shown to be preferentially expressed by skin-resident T-cells for their maintenance of skin homeostasis [61]. It is therefore possible that IgE-binding monocytes in healthy horses readily home to the skin and maintain skin homeostasis through a similar mechanism (Fig. 5). In a prior study on the homeostatic mechanism of CCR10 [61], effector T-cells infiltrating the skin during inflammation did not express CCR10, suggesting that CCR10 is involved in the maintenance of healthy skin, rather than recovery of inflamed skin. Therefore, downregulation of *CCR10* by IgE-binding monocytes in allergic individuals may also reduce skin homing and consequently reduce their contribution to skin homeostasis. Future work needs to address the differences in *CCR10* expression and skin homing of IgE-binding monocytes and skin tissue-associate macrophages in healthy and allergic individuals. Whether allergic inflammation induces the downregulation of *CCR10*, or IgE-binding monocytes in allergic individuals are preprogrammed to express less *CCR10*, remains to be explored.

Together we report both the coagulation cascade, specifically the genes *F13A1*, *SERPINE1* and *C1R*, and the CCR10/CCL27 axis, as candidate pathways used by IgE-binding monocytes in the mechanism of allergy. Significant upregulation of *F13A1* in allergic horses both during clinical allergy and during remission suggests that IgE-binding monocytes may deposit excess fibrin, enhancing allergic dermatitis and skin inflammation. Downregulation of *CCR10* in allergic horses during clinical allergy suggests an impaired ability of IgE-binding monocytes to home to the skin and maintain skin homeostasis. These results highlight specific transcriptional differences that begin to describe the role IgE-binding monocytes play in maintaining allergy, even in the absence of allergen exposure.

## Supplementary information

Supplemental Material

## Data Availability

The datasets generated and analyzed during the current study are available in the NCBI’s Gene Expression Omnibus (GEO) [62] and are accessible through GEO Series accession number GSE231427. All other data generated and analyzed during this study are available from the corresponding author on reasonable request.
