# Supplementary material for: IgE-binding monocytes upregulate the coagulation cascade in allergic horses
Source: Genes Immun. 2023 May 16;24(3):130–8. doi: 10.1038/s41435-023-00207-w (PMC10266973; doi:10.1038/s41435-023-00207-w)
Supplement: Supplementary file 1 — Supplemental Material [file 41435_2023_207_MOESM1_ESM.pdf]

Supplemental Table 1: RNA-seq Sample Quality Control.

Timepoint	Sample	IgE-binding Monocyte viability (%)[a]	RNA concentration (ng/µl)	Fragment Analysis (RQN)
Remission Phase	Allergic 1	100	22.0	9.1
	Allergic 2	100	35.5	8.2
	Allergic 3	98.3	34.3	10
	Allergic 4	95.8	49.3	10
	Allergic 5	100	35.9	10
	Allergic 6	98.5	45.0	9.2
	Allergic 7	100	36.6	10
	Healthy 1	86.7	47.6	10
	Healthy 2	100	44.4	10
	Healthy 3	97.4	44.3	10
	Healthy 4	100	36.7	10
	Healthy 5	100	31.6	10
Clinical Phase	Allergic 1	100	39.5	10
	Allergic 2	100	36.1	9.1
	Allergic 3	98.3	37.9	10
	Allergic 4	95.8	29.6	9.6
	Allergic 5	100	27.1	9.8
	Allergic 6	98.5	38.5	10
	Allergic 7	100	33.7	8.6
	Healthy 1	86.7	29.5	9.9
	Healthy 2	100	34.6	10
	Healthy 3	97.4	33.1	7.6
	Healthy 4	100	34.0	10
	Healthy 5	100	37.7	9.9

a. Cell viability following FACS was measured by Trypan Blue exclusion.

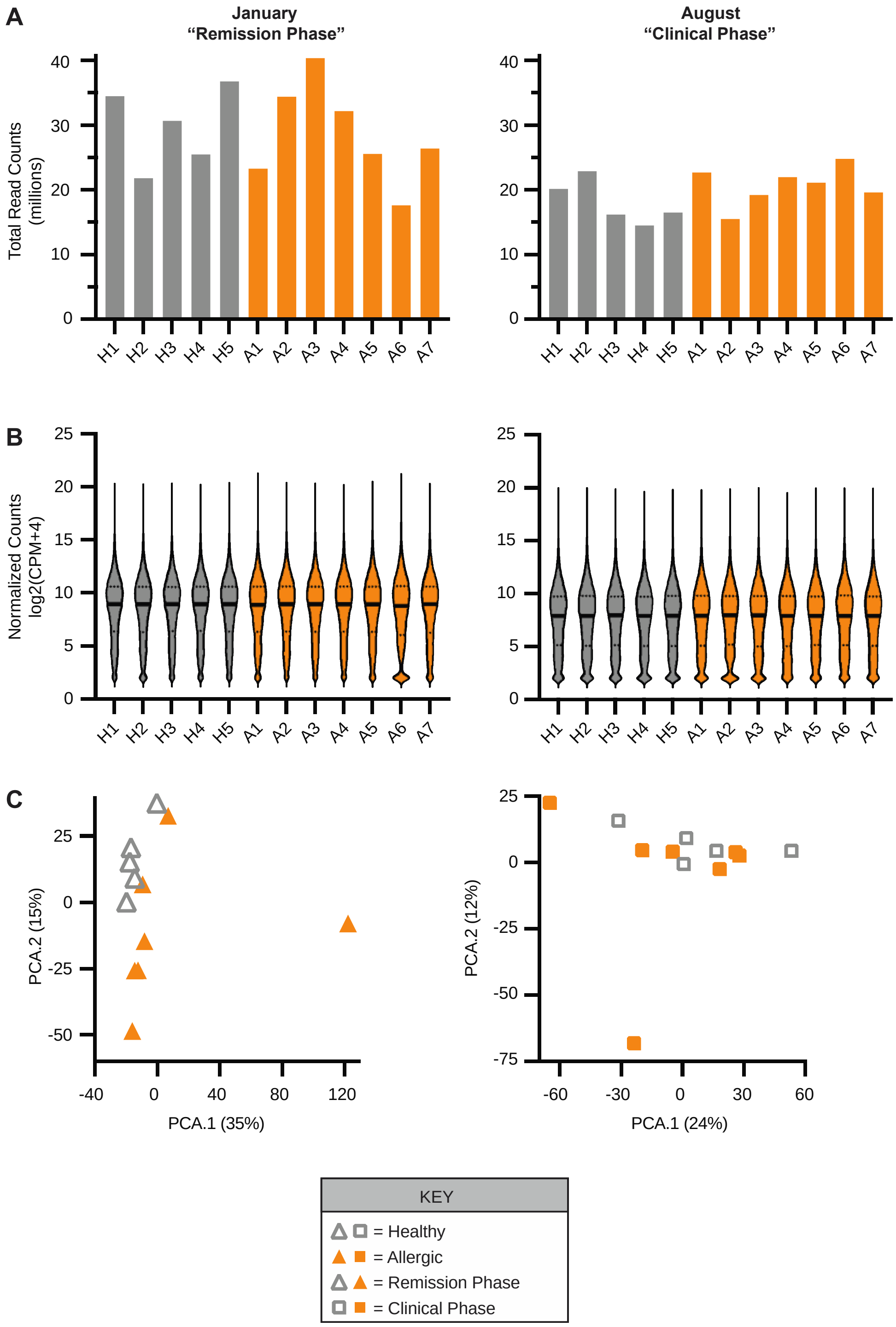

Supplemental Figure 1: Validation of RNA-seq quality.

RNA-seq reads were processed and confirm consistent library quality across all samples. Samples from healthy (n=5, gray bars and △☐) and allergic (n=7, orange bars and ▲■) horses were compared in January (“Remission Phase”, triangles, left graphs) and August (“Clinical Phase”, squares, right graphs). (A) Total read counts from each sample are compared. Bars show sum of read counts for all genes. (B) Normalized expression values for each sample are compared. Violin plots summarize normalized expression for each gene in each sample. Horizontal lines within violin plots show median (solid line) and 1st and 3rd quartiles (dotted lines). (C) Principal Component Analysis (PCA) compares principal components 1 (PCA.1) and 2 (PCA.2). Percentages report variance explained by the corresponding principal component.

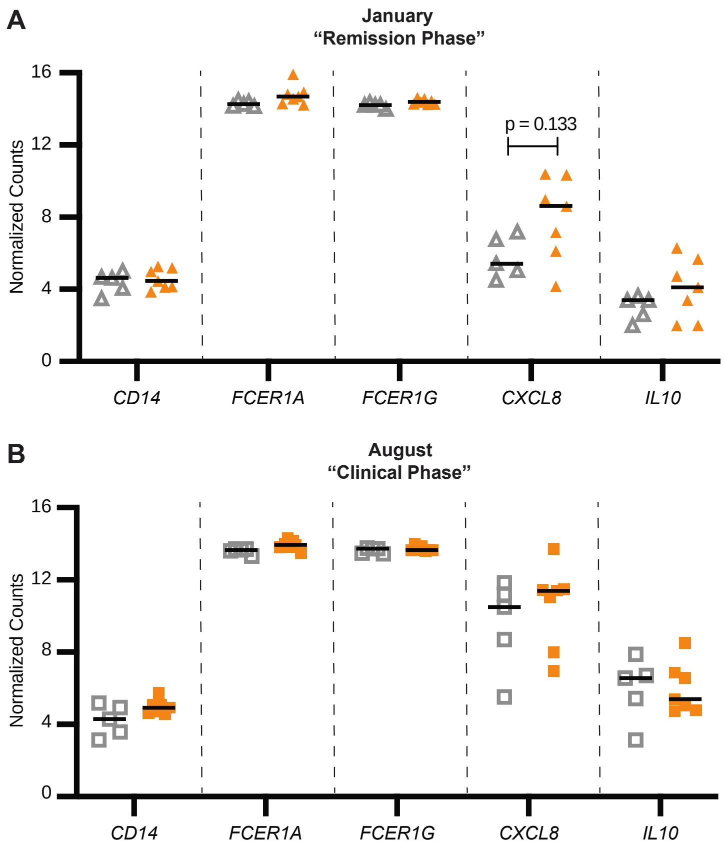

Supplemental Figure 2: IgE-binding monocytes express transcripts for CD14, FcεRI, IL-8 and IL-10 in allergic and healthy horses.

Normalized read counts were compared between healthy (n=5, gray △☐) and allergic (n=7, orange ▲■) horses to confirm identity of IgE-binding monocytes. Expression of genes CD14, FcεRI subunits alpha (*FCER1A*) and gamma (*FCER1G*), IL-8 (*CXCL8*) and IL-10 (*IL10*) were confirmed. (A) Read count comparison in January (“Remission Phase”, triangles). (B) Read count comparison in August (“Clinical Phase”, squares). Graphs plot individual values and medians.

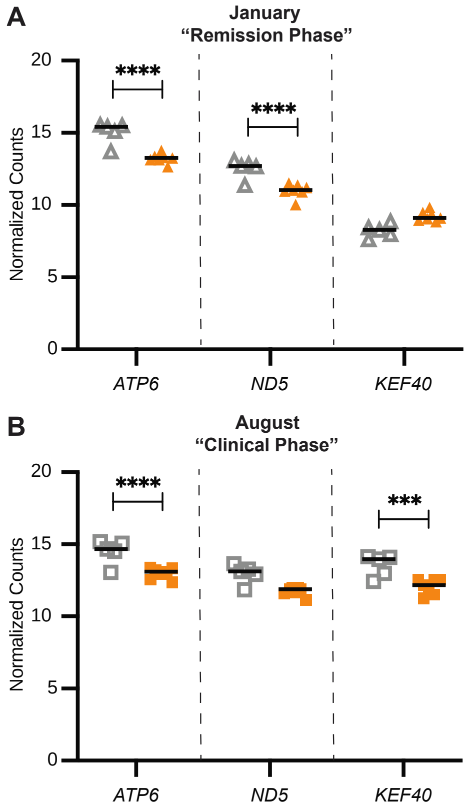

Supplemental Figure 3: Downregulation of mitochondrial genes in IgE-binding monocytes supports a role for decreased immunometabolism in allergic individuals.

Three mitochondrial genes were differentially expressed in allergic horses. Normalized read counts of each differentially expressed mitochondrial gene with greater than -1.5 FC were compared between healthy (n=5, gray △☐) and allergic (n=7, orange ▲■) horses. Downregulation of mitochondrial genes, *ND5*, *KEF40* and *ATP6*, suggest that IgE-binding monocytes in allergic horses have partially impaired metabolic functions. Immunometabolism has been shown to be important in allergies[1] and is worth exploring further. (A) Read count comparison in January (“Remission Phase”, triangles). *ATP6* (-1.9 FC, p=0.0000000091) and *ND5* (-1.6 FC, p=0.000051) were both significantly downregulated during the “Remission Phase”. (B) Read count comparison in August (“Clinical Phase”, squares). *ATP6* was also significantly downregulated during the “Clinical Phase” (-1.6 FC, p=0.000064), and *KEF40* was also downregulated during the “Clinical Phase” (-1.5 FC, p=0.00012). Graphs plot individual values and medians. *** p<0.001, **** p<0.0001.

1. Goretzki A, Lin Y-J, Schülke S. Immune metabolism in allergies, does it matter?—A review of immune metabolic basics and adaptations associated with the activation of innate immune cells in allergy. *Allergy* 2021; **76**: 3314–3331.
